# Composition-Dependent Viscosity and Properties of MCC/CNC Regenerated Cellulose Hydrogels Prepared Using TBAF/DMSO

**DOI:** 10.3390/gels12100878

**Published:** 2026-09-28

**Authors:** Na Hyeon Kim, Maria Jose Silva Pincay, Yeongjun Kim, Hyosub Kim, Eunok Jang, Dong Hyun Kim, Sae-Byuk Lee, Mi-Kyung Park, Seockmo Ku, Eunsook Lee, Deokyeong Choe

**Affiliations:** 1 School of Food Science and Biotechnology, Kyungpook National University, Daegu 41566, Republic of Korea; nanutime@naver.com (N.H.K.); meryjean_s@hotmail.com (M.J.S.P.); ss6277@knu.ac.kr (Y.K.); newriest15@outlook.com (H.K.); eunok021110@naver.com (E.J.); dhkim85@knu.ac.kr (D.H.K.); lsbyuck@knu.ac.kr (S.-B.L.); parkmik@knu.ac.kr (M.-K.P.); 2 Department of Food Science and Technology, Texas A&M University, College Station, TX 77843, USA; seockmo.ku@ag.tamu.edu; 3 Department of Pharmaceutical Science, Florida A&M University, Tallahassee, FL 32307, USA; eunsook.lee@famu.edu

**Keywords:** microcrystalline cellulose, cellulose nanocrystals, tetrabutylammonium fluoride/dimethyl sulfoxide, regenerated cellulose hydrogel, viscosity behavior

## Abstract

Although microcrystalline cellulose (MCC) and cellulose nanocrystals (CNCs) possess the same cellulose backbone, it remains unclear whether they have similar effects on solution viscosity behavior in tetrabutylammonium fluoride (TBAF)/dimethyl sulfoxide (DMSO) and the properties of the resulting regenerated hydrogels. This study investigated the composition-dependent viscosity behavior of MCC/CNC solutions in TBAF/DMSO and the properties of the regenerated cellulose hydrogels. At 2.5% cellulose, the MCC solution achieved a maximum viscosity of 8.8 Pa·s at 2.5% TBAF, whereas the CNC solution reached 15.0 Pa·s at 5.5% TBAF. In mixed solutions, increasing the CNC content decelerated the viscosity increase, extending the time required for solution preparation and molding. After water-induced regeneration, the gel strength and cutting strength decreased from 70.8 to 33.6 N·mm and from 55.8 to 6.7 N·mm, respectively, as the formulation shifted from MCC-only to CNC-only. Conversely, the CNC-containing hydrogels exhibited higher optical transmittance than MCC-only hydrogels. Time-resolved photoluminescence measurements suggested composition-dependent local photophysical relaxation. Fourier-transform infrared spectroscopy revealed no formation of new covalent structures, while X-ray diffraction indicated that the regenerated hydrogels were predominantly amorphous. Thermogravimetric analysis revealed composition-dependent thermal degradation. These results demonstrate that controlling the MCC/CNC compositional ratio provides a means of balancing solution processability and regenerated hydrogel properties.

## 1. Introduction

Cellulose is one of the most abundant natural polymers on Earth, and it has been widely investigated for various material applications, including hydrogels, films, membranes, packaging materials, and biomedical materials, owing to its renewability, biodegradability, biocompatibility, and excellent mechanical properties [1,2,3]. In particular, cellulose hydrogels offer considerable potential as environmentally friendly functional materials because they combine a three-dimensional network structure with a high water content [4,5]. Recent studies have also highlighted cellulose-based hydrogels as sustainable biomaterials with tunable mechanical, biological, chemical, and surface properties for biomedical and tissue-engineering applications [6]. However, cellulose is poorly soluble in common solvents because of its highly ordered crystalline structure and strong interchain associations, which are stabilized by extensive intramolecular and intermolecular hydrogen bonding, van der Waals/dispersion interactions, and hydrophobic associations [7,8,9].

To overcome this limitation, several nonderivatizing cellulose solvents, including lithium chloride/*N*,*N*-dimethylacetamide, sodium hydroxide/urea, and ionic liquids, have been developed and employed for cellulose dissolution and regeneration [10,11,12]. Although these solvents are effective under their respective processing conditions, their use may involve requirements or limitations, such as cellulose activation, strict temperature control, moisture sensitivity, high solution viscosity, and solvent recovery challenges [12,13]. Tetrabutylammonium fluoride (TBAF)/dimethyl sulfoxide (DMSO) also has limitations, including the dependence of cellulose dissolution on the water content and composition of the TBAF/DMSO solvent system, as well as limited solvent recyclability [14,15]. Nevertheless, TBAF/DMSO is a useful system for investigating composition-dependent solution viscosity and subsequent cellulose regeneration because it has been reported to dissolve cellulose rapidly at room temperature without prior activation while preserving the degree of polymerization (DP) [16,17].

During cellulose dissolution and regeneration using such nonderivatizing solvents, both the structural characteristics of the starting cellulose material and the viscosity behavior of the cellulose solution can influence the final properties of the regenerated cellulose hydrogels [4,18,19]. Generally, the viscosity of polymer solutions is known to strongly depend on polymer concentration and molecular weight [20]. Therefore, controlling the viscosity of a cellulose solution by adjusting the solvent or ionic composition while maintaining the same polymer concentration and DP can have important processing implications for cellulose hydrogel fabrication.

In our previous study, we controlled the viscosity behavior of cellulose solutions by dissolving microcrystalline cellulose (MCC) in TBAF/DMSO and varying only the TBAF concentration while maintaining a constant MCC concentration [19]. A unique phenomenon was observed in which the viscosity of the MCC solution increased sharply at a specific TBAF concentration. This behavior was attributed to changes in cellulose–cellulose interactions involving both individual cellulose chains and aggregates of cellulose chains in TBAF/DMSO, together with the possible formation of a liquid-crystalline phase. In addition, regenerated cellulose hydrogels prepared from high-viscosity cellulose solutions exhibited high mechanical strength, and a strong positive correlation was observed between solution viscosity and hydrogel strength. These results suggest that controlling the viscosity of cellulose solutions in TBAF/DMSO may provide a useful strategy for regulating the properties of regenerated cellulose hydrogels.

However, it remains unclear whether the observed TBAF concentration-dependent viscosity behavior is specific to MCC or can also occur in cellulose sources with different structural characteristics. MCC and cellulose nanocrystals (CNCs) share the same cellulose backbone but differ markedly in particle size, specific surface area, crystallinity, and surface chemistry [21,22]. MCC consists of micrometer-scale cellulose particles, whereas CNCs are typically prepared through sulfuric acid hydrolysis, which removes amorphous cellulose regions and yields nanoscale particles with high specific surface areas and sulfate-bearing anionic surfaces [21,23,24]. These structural differences may affect how each cellulose source interacts with the solvent and develops viscosity in TBAF/DMSO. Moreover, CNCs are known to act as rigid nanofillers or reinforcing phases in composite materials because of their high crystallinity and rigidity [22,25,26]. Nevertheless, it is not yet established whether MCC and CNCs influence solution behavior and the properties of the regenerated hydrogels similarly during TBAF/DMSO-based cellulose regeneration. Therefore, rather than simply treating MCC and CNCs as equivalent cellulose sources, it is necessary to determine whether they exhibit distinct viscosity behavior in TBAF/DMSO and whether this difference is reflected in the properties of the resulting regenerated hydrogels. Clarifying these composition-dependent effects is important for the development of soft, transparent, and mechanically tunable regenerated hydrogels, as it requires control over both solution processability and the properties of the final hydrogel [27,28].

In the present study, we systematically investigated how the structural differences between MCC and CNC affect the viscosity behavior of cellulose solutions in TBAF/DMSO and the properties of regenerated hydrogels. To this end, MCC and CNCs were individually treated at a fixed cellulose concentration with varying TBAF concentrations to compare their component-specific viscosity behavior. Subsequently, MCC/CNC solutions with various compositional ratios were prepared in TBAF/DMSO, and their time-dependent viscosity increase was evaluated. Regenerated MCC/CNC hydrogels were then prepared from each solution, and their composition-dependent physical, optical, chemical, structural, thermal, and morphological properties were compared using gel-strength and cutting-strength measurements, optical transmittance, time-resolved photoluminescence (TRPL) relaxation behavior, Fourier-transform infrared (FT-IR) spectroscopy, X-ray diffraction (XRD), scanning electron microscopy (SEM), and thermogravimetric analysis (TGA) with derivative thermogravimetry (DTG).

The objective of this study was to clarify how the MCC/CNC compositional ratio regulates the viscosity behavior of cellulose solutions in TBAF/DMSO and the properties of regenerated hydrogels. Specifically, this study aimed to determine whether CNCs act simply as rigid reinforcing nanofillers or instead undergo structural disruption and contribute to the formation of predominantly amorphous regenerated hydrogels during TBAF/DMSO-based cellulose regeneration. This approach enabled us to elucidate the contrasting effects of formulations with high MCC ratios and high CNC ratios on solution processability, optical homogeneity, mechanical resistance, and thermal degradation behavior, thereby providing a useful basis for the compositional design of regenerated cellulose hydrogels processed using TBAF/DMSO.

## 2. Results and Discussion

### 2.1. Viscosity Behavior and Control of MCC/CNC Solutions in TBAF/DMSO

In TBAF/DMSO, both MCC and CNCs exhibited a characteristic viscosity response, where their viscosity increased as the TBAF concentration increased and then decreased at higher TBAF concentrations (Figure 1). However, the two cellulose sources required substantially different TBAF concentrations to attain maximum viscosity. When the total cellulose concentration was fixed at 2.5%, the viscosity of the MCC solution increased with increasing TBAF concentration, reached a maximum value of approximately 8.8 Pa·s at 2.5% TBAF, and then decreased at higher TBAF concentrations (Figure 1a). The viscosity of the CNC solution also increased and subsequently decreased as a function of the TBAF concentration, reaching a maximum value of approximately 15.0 Pa·s at 5.5% TBAF (Figure 1b). These results indicate that although MCC and CNCs display a similar concentration-dependent viscosity pattern in TBAF/DMSO, the TBAF concentration required to achieve maximum viscosity differs between the two cellulose sources, which may be related to differences in their structural characteristics.

Although MCC and CNCs share the same cellulose backbone, their different viscosity responses in TBAF/DMSO may be associated with differences in structural characteristics, including particle size, specific surface area, DP, and surface characteristics. The MCC used in this study had a DP of 186, whereas the CNCs had an estimated DP-equivalent range of approximately 88–168 based on the molecular formula and molecular weight provided by the manufacturer. Although the upper end of the estimated CNC DP range approaches the DP of MCC, the range also extends to substantially lower values; it must be noted that the actual DP of the CNCs used in this study was not determined. Thus, differences in chain length may have contributed to the observed viscosity behavior, but DP alone is insufficient to fully explain the viscosity response of the CNC solution. Under the present TBAF/DMSO conditions, the relatively large particle size and low specific surface area of MCC may have limited its interactions with TBAF/DMSO and the extent of structural disruption, potentially resulting in less extensive aggregate dissociation and the persistence of relatively large cellulose aggregates. In contrast, the nanoscale dimensions and high specific surface area of the CNCs may have facilitated their interactions with TBAF/DMSO and promoted more extensive structural disruption and aggregate dissociation. Consequently, the CNC solution may have contained a relatively larger population of more highly dispersed cellulose chains and smaller cellulose aggregates. These species may participate more effectively in transient solution-state interactions, contributing to the observed apparent viscosity without necessarily requiring complete molecular dissolution.

It is also noteworthy that the CNC solution reached its maximum viscosity at a higher TBAF concentration (5.5%) than the MCC solution. In TBAF/DMSO, fluoride ions can weaken the hydrogen-bonding network associated with the hydroxyl groups or acetal oxygen atoms of cellulose and contribute to the disruption of the cellulose structure [14]. However, because CNCs possess high initial crystallinity, sulfate-bearing structures, and a hydration/ionic environment, their effective interactions with TBAF/DMSO may differ from those of MCC. Therefore, a higher nominal TBAF concentration may be required to sufficiently disrupt the CNC structure. At 5.5% TBAF, a balance between structural disruption, aggregate dissociation, and transient associations involving cellulose chains and their aggregates may result in the highest apparent viscosity. In our previous study on MCC/TBAF/DMSO [19], viscosity also decreased when the TBAF concentration exceeded the concentration associated with the maximum viscosity. This decrease was attributed to excess ions weakening cellulose–cellulose associations. A similar explanation may apply to the CNC solution: at higher TBAF concentrations, further structural disruption and solvation, together with ionic screening and reduced cellulose–cellulose association, may have contributed to the observed viscosity decrease. Figure 1c presents a schematic comparison of the proposed relative differences in structural disruption, aggregate dissociation, and dispersed chain populations between the MCC and CNC solutions under their respective maximum-viscosity conditions.

The time-dependent viscosity changes of TBAF/DMSO solutions containing both MCC and CNCs were then examined (Figure 1d). The MCC-only solution exhibited the most rapid viscosity increase and reached 10 Pa·s first. Conversely, as the CNC content and the corresponding nominal TBAF concentration increased across the formulation series, the time required to reach 10 Pa·s gradually increased. The CNC-only solution exhibited the slowest viscosity increase. This result suggests that the introduction of CNCs, under the corresponding TBAF conditions used in this study, can modulate the rate of viscosity increase in MCC/CNC solutions. In particular, the rapid viscosity buildup of the MCC-only solution can cause the solution to thicken within a short period, making homogeneous mixing and shaping difficult. Such rapid viscosity increase may result in local compositional heterogeneity, incomplete mixing, difficulties in mold filling, and irregular hydrogel shapes during solution preparation. These characteristics can limit reproducibility and shape control during hydrogel fabrication. In contrast, the slower viscosity increase observed in formulations with elevated CNC content may provide additional time for more homogeneous mixing and shaping of MCC/CNC solutions, thereby offering a processing advantage. In this regard, CNC-containing formulations may expand the processing window during which cellulose components can be dispersed and undergo structural disruption in TBAF/DMSO, thereby facilitating more homogeneous solution preparation, stable mold filling, and reproducible hydrogel fabrication.

It should be noted that no viscosity plateau was experimentally confirmed for any formulation because the viscosity continued to increase beyond the reliable measurement range of the viscometer at the applied shear rate. Therefore, the time required to reach a plateau could not be determined. The value of 10 Pa·s shown in Figure 1d was used solely as a common reference viscosity to compare the rates of viscosity increase among the formulations. It was not considered as a gelation threshold or a fixed limit for processability. Additionally, the processing window in this study refers to the practical period after solution preparation during which the mixture remains sufficiently fluid for homogeneous mixing, transfer, and mold filling before rapid viscosity buildup restricts these operations. No fixed viscosity threshold was used to define this period. Importantly, the trends observed in the mixed formulations should not be interpreted as independent effects of CNC content. The nominal TBAF concentration varied systematically with the MCC/CNC compositional ratio in these formulations, accompanied by changes in ionic and water content. Thus, these coupled changes should be considered when interpreting the observed trends.

Overall, the results presented in Figure 1 demonstrate that the viscosity behavior of MCC/CNC–TBAF/DMSO solutions can be effectively controlled through formulation design involving adjustments of the MCC/CNC compositional ratio and the corresponding TBAF concentration. Although differences in DP may contribute to the viscosity behavior, DP alone cannot fully account for the observed viscosity response of the CNC solution. The nanoscale dimensions and high specific surface area of CNCs may also facilitate their interactions with TBAF/DMSO and promote structural disruption and the participation of more highly dispersed cellulose chains and smaller cellulose aggregates in solution-state interactions. In addition, CNC-containing formulations delay the rapid viscosity buildup observed in the MCC-only solution, thereby providing a broader processing window for solution preparation and shaping.

### 2.2. Mechanical Properties of Regenerated Hydrogels as a Function of MCC/CNC Compositional Ratio

As noted above, the time-dependent increase in apparent viscosity may reflect progressive structuring of the cellulose solutions prior to the self-supporting state observed after overnight standing. Although we did not directly characterize the specific mechanism and rheological transition associated with formation of the intermediate gels under the present formulation conditions, previous work has demonstrated that gelation of cellulose in TBAF/DMSO can be promoted by interactions between water and fluoride ions [14]. These interactions reduce the association of fluoride ions with cellulose and allow cellulose–cellulose hydrogen bonds to reform, thereby promoting chain reassociation. This previously reported mechanism provides a plausible mechanistic context for the observed formation of the self-supporting intermediate gels; nonetheless, its direct involvement under the present conditions was not established. After water-induced solvent exchange and regeneration, the gel strength and cutting strength of the resulting hydrogels were compared across the different MCC/CNC compositional ratios (Figure 2). The MCC-only hydrogel exhibited the highest gel strength; however, as the CNC content increased, the gel strength generally decreased (Figure 2a). A similar trend was observed for cutting strength: hydrogels prepared with high MCC ratios displayed relatively high values, while those prepared with high CNC ratios exhibited reduced cutting strength (Figure 2b). These results indicate that the MCC/CNC compositional ratio is associated with differences in the mechanical resistance of hydrogels formed after regeneration in water.

The high gel strength and cutting strength of the MCC-only hydrogel may be attributed to the formation of an effective load-bearing network by MCC-derived cellulose chains or their aggregates during water-induced regeneration. Differences in chain length possibly contributed to this behavior, as the estimated DP-equivalent range of the CNCs extends to values substantially lower than the measured DP of MCC. Longer cellulose chains can favor network connectivity, chain bridging, and entanglement formation after partial structural disruption or dissolution in TBAF/DMSO followed by regeneration. Additionally, cellulose–cellulose hydrogen-bonded junctions formed during water-induced regeneration may have provided stronger mechanical resistance in the formulations with high MCC ratios.

Conversely, the hydrogels with high CNC contents exhibited decreases in both gel strength and cutting strength. Potential differences in chain length could have contributed to the reduced long-range connectivity of the cellulose network formed from the CNCs during regeneration. However, differences in DP alone may be insufficient to fully account for the observed mechanical behavior. Moreover, because CNCs contain sulfate-bearing structures and a hydration/ionic environment, the formation of dense and strong junctions based on cellulose–cellulose hydrogen bonding may have been limited during water-induced regeneration. Therefore, although the formulations with high CNC ratios exhibited a slower viscosity increase and improved processability in the TBAF/DMSO solution state, they appeared to form relatively weaker load-bearing hydrogel networks after water-induced regeneration compared to the formulations with high MCC ratios.

Because the mechanical properties of hydrogels can be influenced by their hydration state, the water content of the regenerated hydrogels was evaluated. The water content increased significantly as the CNC content increased. Specifically, it increased from 95.22% ± 0.05% for the MCC-only hydrogel (MCC:CNC = 10:0) to 97.97% ± 0.19% for the CNC-only hydrogel (MCC:CNC = 0:10) (Table S1). The intermediate formulations displayed water contents of 96.18% ± 0.05%, 96.77% ± 0.21%, and 97.42% ± 0.20% at MCC:CNC ratios of 7.5:2.5, 5:5, and 2.5:7.5, respectively. These results indicate that the water content of the regenerated hydrogels varied systematically with the MCC/CNC composition. The increase in water content associated with elevated CNC contents may have contributed to the lower mechanical resistance. Collectively, the observed mechanical behavior likely reflects the combined effects of composition-dependent differences in water content and cellulose-network formation rather than a single structural factor.

These results emphasize that the time-dependent viscosity increase in the solution state and mechanical strength after hydrogel regeneration do not necessarily change in the same direction. As shown in Figure 1, the CNC-containing formulations delayed the rapid viscosity buildup observed in the MCC-only solution and provided a broader processing window; however, as depicted in Figure 2, the gel strength and cutting strength of the hydrogels decreased as the CNC content increased. Overall, formulations with high MCC ratios are favorable for forming strong load-bearing networks after regeneration, whereas formulations with high CNC ratios exhibit a slower viscosity increase and may offer improved processability during solution preparation. Therefore, the MCC/CNC compositional ratio serves as an important formulation parameter associated with both solution-state processability and the mechanical properties of regenerated hydrogels under the corresponding TBAF conditions.

### 2.3. Optical Properties and TRPL Relaxation Behavior of Regenerated MCC/CNC Hydrogels

To examine the optical properties of the regenerated MCC/CNC hydrogels as a function of composition, the apparent transparency of the hydrogels placed on a printed background was compared, and their ultraviolet–visible (UV–Vis) transmittance spectra were recorded. As shown in Figure 3a, all regenerated hydrogels exhibited generally high apparent transparency, as evidenced by the visibility of the printed background through the hydrogel samples. This apparent transparency is consistent with the high water content of the hydrogel networks.

Although the regenerated hydrogels appeared equally transparent when observed by the naked eye, the UV–Vis transmittance results revealed substantial differences in optical transmittance depending on the MCC/CNC composition (Figure 3b). Hydrogels prepared with high MCC ratios displayed relatively lower transmittance, whereas those prepared with high CNC ratios exhibited generally higher transmittance. These results suggest that even though all hydrogels appeared transparent, the uniformity of the internal cellulose network and the degree of light scattering varied depending on the MCC/CNC compositional ratio. The high water contents observed at increasing CNC ratios may also have contributed to the higher optical transmittance.

The lower transmittance of the hydrogels prepared with high MCC ratios may be attributed to the formation of relatively large, nonuniformly regenerated regions during water-induced regeneration, which could increase light scattering. As shown in Figure 1, the MCC-only solution underwent rapid viscosity buildup, which may have limited the time available for uniform mixing and molding. This rapid increase in viscosity could have promoted local compositional heterogeneity or the formation of larger scattering regions during regeneration, thereby reducing UV–Vis transmittance. In contrast, the CNC-containing formulations exhibited a slower viscosity increase, providing a broader processing window. Additionally, the nanoscale structure and high specific surface area of the CNCs may have enabled more uniform dispersion and greater structural disruption in TBAF/DMSO. Consequently, the hydrogels prepared with high CNC ratios exhibited reduced light scattering, which resulted in higher optical homogeneity and transmittance.

To further examine the effect of the MCC/CNC compositional ratio on the photophysical relaxation behavior of the regenerated hydrogels, local excited-state relaxation was compared using TRPL parameters (Figure 3c). Previous studies on cellulose-based cluster luminescence have demonstrated that hydrogen-bonded and restricted local environments can suppress molecular motion and vibrational relaxation, thereby reducing nonradiative decay and affecting emission lifetimes [29,30]. In particular, stepwise rigidification of cellulose has been proposed to suppress nonradiative decay and extend phosphorescence lifetimes, while rigid microenvironments formed by hydrogen bonding and cross-linking have been reported to influence excited-state relaxation pathways [29]. In addition, dense O–H···O hydrogen-bond networks in cellulose nanospheres have been reported to form restricted microenvironments that suppress vibrational relaxation and reduce nonradiative decay channels [30].

In this study, as the CNC content increased, the amplitude-weighted average lifetime (τ_amp) decreased, while the fractional contribution of the fast decay component (B_1_) increased (Figure 3c). These changes are consistent with faster excited-state relaxation in the hydrogels prepared with higher CNC ratios. Considering that restricted hydrogen-bonded environments in cellulose-based luminescent systems can suppress molecular motion and nonradiative relaxation, these results may indicate a relatively less restricted local photophysical environment in these hydrogels. It is worth noting that the TRPL results provide complementary evidence of composition-dependent local photophysical behavior and should therefore not be interpreted as a direct measure of bulk hydrogel performance.

Overall, the results presented in Figure 3 indicate that both the optical homogeneity and local photophysical relaxation behavior of the regenerated hydrogels varied with the MCC/CNC compositional ratio. Hydrogels prepared from CNC-containing formulations exhibited reduced light scattering relative to those prepared from formulations with high MCC ratios, thereby resulting in higher optical transmittance. Furthermore, the decrease in τ_amp and increase in B_1_ observed in the TRPL analysis were consistent with composition-dependent differences in local photophysical relaxation in the regenerated hydrogels.

### 2.4. Chemical, Structural, Thermal, and Morphological Characteristics of the Regenerated MCC/CNC Hydrogels

FT-IR spectroscopy, XRD, TGA/DTG, and SEM analyses were performed to examine the chemical structure, crystalline structure, thermal degradation behavior, and morphology of the regenerated MCC/CNC hydrogels (Figure 4 and Figure 5). The FT-IR spectra of all regenerated hydrogels exhibited the major absorption bands associated with cellulose (Figure 4a). Specifically, a broad O–H stretching band was observed in the 3700–3000 cm^−1^ region, together with characteristic bands near 2900 cm^−1^ (C–H/CH_2_ stretching), 1639 cm^−1^ (absorbed water), 1376 cm^−1^ (C–H deformation), 1163 cm^−1^ (C–O–C pyranose-ring skeletal vibration), and 897 cm^−1^ (β-glucosidic linkage). These observations are consistent with previously reported FT-IR spectra of regenerated cellulose [11,31]. These characteristic absorption bands appeared at similar wavenumbers across all compositions, and no clear composition-dependent shifts or distinct new absorption bands were observed with changes in the MCC/CNC composition. These results indicate that the major chemical structure of the cellulose backbone was retained after TBAF/DMSO treatment and water-induced regeneration and that the composition-dependent differences in hydrogel properties were not associated with the formation of new covalent chemical structures.

The XRD patterns were used to evaluate changes in the crystalline characteristics associated with TBAF/DMSO treatment and subsequent water-induced regeneration (Figure 4b and Figure S1). The XRD patterns of the same starting MCC and CNC materials previously reported in Refs. [19] and [3], respectively, displayed distinct cellulose crystalline reflections, with crystallinity indices of 73% and 70%, respectively (Figure S1). In contrast, the regenerated hydrogels exhibited no distinct crystalline peaks across all compositions but instead displayed broad amorphous features (Figure 4b). Because the XRD patterns of the starting materials and regenerated hydrogels were acquired using different diffractometers, they were not compared quantitatively. Nevertheless, the qualitative transition from distinct crystalline reflections in the starting materials to broad diffraction features in the regenerated hydrogels indicates extensive disruption of the initial crystalline structures of the MCC and CNCs during TBAF/DMSO treatment and water-induced regeneration. The regenerated hydrogels were therefore predominantly amorphous.

In particular, the original crystalline character of the CNCs was not retained even in the hydrogels prepared with high CNC ratios, demonstrating that the initial crystalline structure of the CNCs was effectively disrupted after TBAF/DMSO treatment and water-induced regeneration. In general, CNCs are known to act as a reinforcing phase in composite materials because of their high crystallinity and rigidity [22,25,26]. However, under the TBAF/DMSO-based regeneration conditions used in this study, the CNCs did not remain intact as crystalline nanofillers that provided reinforcement. Instead, the CNCs likely underwent structural disruption during TBAF/DMSO treatment and did not retain their original crystalline nanofiller structure after water-induced regeneration. This also aligns with the pronounced TBAF concentration-dependent viscosity response observed for the CNC solution (see Figure 1). In other words, the nanoscale structure and high specific surface area of the CNCs may have promoted structural disruption in TBAF/DMSO, increasing the effective population of cellulose chains and their aggregates capable of contributing to the viscosity response. The loss of CNC crystalline character confirmed by XRD supports this possibility.

Figure 4c shows the TGA curves and DTG profiles of the regenerated MCC/CNC hydrogels. All samples exhibited an initial weight loss in the low-temperature region, which may be associated with the removal of residual water or weakly bound water remaining in the regenerated hydrogels or freeze-dried samples. The main cellulose degradation occurred approximately between 200 and 400 °C; however, the degradation profiles changed markedly depending on the MCC/CNC compositional ratio. The MCC-only hydrogel exhibited rapid weight loss over a relatively higher temperature region and displayed a single, distinct, and sharp main degradation peak in its DTG profile. This indicates that the MCC-derived regenerated cellulose network underwent concentrated thermal decomposition within a relatively narrow temperature range. Conversely, as the CNC content increased, the main weight-loss region tended to shift toward lower temperatures, and the main degradation peak in the DTG profile also gradually shifted to a lower-temperature region. In particular, the hydrogels prepared with high CNC ratios exhibited broader DTG peaks than the MCC-only hydrogel, along with shoulder-like features or distributed degradation signals. These results indicate that increasing CNC content transforms the thermal degradation of regenerated MCC/CNC hydrogels from a single, concentrated decomposition event into composition-dependent multiple degradation processes.

The lower-temperature shift and broadened DTG features observed for the hydrogels prepared with high CNC ratios may be related to the structural characteristics of the CNC-derived cellulose. The CNCs used in this study had an estimated DP-equivalent range that extended below the DP of MCC and contained sulfate-bearing structures as well as a hydration/ionic environment. It has been reported that cellulose crystals or CNCs bearing sulfate groups can begin thermal degradation at relatively low temperatures and that their degradation reactions can be separated into low-temperature and high-temperature processes in DTG profiles [24,32]. Therefore, the lower-temperature shift of the main degradation peak and the broadened/multistep DTG behavior observed in the hydrogels prepared with high CNC ratios may be associated with the combined effects of CNC-derived sulfate-bearing cellulose, potential differences in chain length, higher surface accessibility, and the predominantly amorphous character of the regenerated hydrogels.

SEM was additionally used to examine the surface and cross-sectional morphologies of the freeze-dried regenerated MCC/CNC hydrogels (Figure 5). The five formulations exhibited broadly similar morphological features, albeit with some local variations. No clear or systematic changes in surface or cross-sectional morphology were evident with increasing CNC content at the examined magnifications. These observations suggest that variation in the MCC/CNC composition did not result in pronounced differences in the gross morphology detectable by SEM.

Overall, the combined FT-IR, XRD, TGA/DTG, and SEM results demonstrate that the regenerated MCC/CNC hydrogels maintained their cellulose backbone after TBAF/DMSO treatment and water-induced regeneration, while the initial crystalline structures of both MCC and CNCs were substantially disrupted, yielding regenerated hydrogels with predominantly amorphous characteristics. In addition, SEM revealed no pronounced composition-dependent differences in gross morphology. In contrast, variations in the MCC/CNC compositional ratio were associated with differences in the thermal degradation pathways of the regenerated cellulose rather than with the formation of new covalent chemical structures. In particular, the lower-temperature shift of the DTG peak and the broadened or multistep degradation profiles observed with high CNC ratios suggest that CNC-derived sulfate-bearing cellulose may contribute to the composition-dependent thermal degradation behavior.

## 3. Conclusions

This study demonstrates that MCC and CNCs should not be regarded as equivalent cellulose sources for TBAF/DMSO-based cellulose regeneration. Although both cellulose sources exhibited TBAF concentration-dependent viscosity behavior, their roles in solution preparation and regenerated hydrogel formation were clearly different. The formulations with high MCC ratios favored the formation of mechanically resistant hydrogel networks after water-induced regeneration, whereas CNC-containing formulations delayed the rapid viscosity buildup observed in the MCC-only solution, thereby broadening the processing window for solution preparation and molding. As the CNC content increased, the gel strength and cutting strength decreased, indicating that the nanoscale dimensions and high surface accessibility of CNCs do not necessarily lead to mechanical reinforcement after regeneration. Instead, the disappearance of crystalline peaks in the XRD patterns indicates that the initial crystalline character of the CNCs was not retained and that the regenerated hydrogels were predominantly amorphous. This structural transition is consistent with the contrasting behavior of the hydrogels prepared with high CNC ratios, which exhibited improved optical transmittance but reduced mechanical resistance and altered thermal degradation profiles. Overall, the MCC/CNC compositional ratio serves as an important formulation parameter associated with both solution-state processability and final hydrogel properties. Formulations with high MCC ratios are advantageous when mechanical resistance is prioritized, whereas CNC-containing compositions are useful when a slow viscosity increase, improved moldability, and optical homogeneity are desired. These findings provide a useful basis for designing regenerated cellulose hydrogels from mixed cellulose sources by considering both TBAF/DMSO-induced structural disruption and water-regeneration-driven network formation. An important remaining knowledge gap is the independent contribution of the MCC/CNC compositional ratio and TBAF concentration, because these two formulation variables were varied simultaneously in the mixed formulations used in this study. In addition, the effect of total cellulose concentration was not systematically examined because it was fixed at 2.5%. Future studies should therefore independently vary the MCC/CNC compositional ratio, TBAF concentration, and total cellulose concentration. Such studies should also determine the critical gelation concentrations of MCC- and CNC-based formulations under defined TBAF/DMSO conditions and further characterize the solution-state organization and rheological behavior of cellulose using rheological instrumentation with a wider measurement range to evaluate shear-rate-dependent viscosity, oscillatory rheology (G′ and G″), and yield stress. These measurements could clarify the relationships among structural disruption, aggregate dissociation, viscosity behavior, and subsequent hydrogel formation.

## 4. Materials and Methods

### 4.1. Materials

MCC (Avicel PH-101, Merck Supelco, Cork, Ireland) was used as the microscale cellulose source. According to the certificate of analysis, the DP of MCC was 186, which corresponded to an estimated molecular weight of approximately 30.2 kDa based on the anhydroglucose unit. The manufacturer reports a nominal mean particle size of approximately 50 μm for Avicel PH-101. CNCs (CelluForce NCV100, CelluForce, Montreal, QC, Canada) were used as the nanoscale cellulose source. According to the manufacturer’s specification, the molecular weight of the CNCs was 14.7–27.85 kDa, which corresponded to an estimated DP-equivalent range of approximately 88–168 based on the manufacturer-provided molecular formula. The manufacturer also reports nominal dimensions of approximately 7.5 nm × 150 nm for individual CNCs and a dry-powder particle size of 10–30 μm. Particle size was not independently measured in this study.

Tetrabutylammonium fluoride trihydrate (TBAF·3H_2_O, ≥97.0%, Sigma-Aldrich, St. Louis, MO, USA) and DMSO (≥99.0%, Tokyo Chemical Industry, Tokyo, Japan) were used for cellulose dissolution. Distilled water was used for cellulose regeneration and solvent exchange. All chemicals and reagents were used as received without further purification.

### 4.2. Preparation of the MCC/CNC Solutions and Regenerated Hydrogels

To compare the viscosity behavior of MCC and CNCs in TBAF/DMSO, viscosity screening was first conducted for each cellulose source by varying the TBAF concentration while maintaining the cellulose concentration at 2.5%. Unless otherwise stated, the MCC, CNC, and TBAF concentrations were calculated based on the volume of DMSO. Thus, a cellulose concentration of 2.5% corresponds to 2.5 g of cellulose per 100 mL of DMSO.

For the MCC solutions, TBAF/DMSO solvents with different TBAF concentrations were first prepared, after which MCC powder was added at a concentration of 2.5%. For the CNC solutions, the same procedure was followed by preparing TBAF/DMSO solvents with different TBAF concentrations and adding CNC powder at a concentration of 2.5%. Immediately after cellulose powder addition, each sample was sufficiently vortexed to facilitate uniform dispersion of the cellulose and its interaction with TBAF/DMSO.

Based on the viscosity screening results, the MCC solution exhibited its maximum viscosity in 2.5% TBAF/DMSO, whereas the CNC solution demonstrated its maximum viscosity in 5.5% TBAF/DMSO. Therefore, 2.5% and 5.5% TBAF/DMSO were selected as the respective maximum-viscosity conditions for the MCC and CNC materials when preparing the formulations with different MCC/CNC compositional ratios. For each mixed formulation, the nominal TBAF concentration was determined by the relative volumes of the 2.5% and 5.5% TBAF/DMSO solvent components and therefore varied systematically with the MCC:CNC compositional ratio. These intermediate nominal TBAF concentrations were not independently optimized to determine the maximum viscosity for each mixed MCC/CNC composition.

The MCC/CNC solutions were prepared at MCC:CNC compositional ratios of 10:0, 7.5:2.5, 5:5, 2.5:7.5, and 0:10. The total cellulose concentration was fixed at 2.5% for all compositions to maintain consistency with our previous study on MCC/TBAF/DMSO hydrogels [19] and to compare the effects of MCC/CNC composition at a constant total cellulose loading. Each MCC/CNC solution was not prepared by separately preparing complete MCC and CNC solutions followed by post-mixing. Instead, 2.5% TBAF/DMSO and 5.5% TBAF/DMSO were first mixed according to the target MCC:CNC ratio, and then MCC and CNC powders were added simultaneously to the mixed TBAF/DMSO solvent. This preparation sequence was used because MCC powder caused a rapid increase in viscosity and formed a gel-like state immediately after being added to TBAF/DMSO, thereby hindering homogeneous post-mixing of separately prepared MCC and CNC solutions.

For example, to prepare a solution with an MCC:CNC ratio of 5:5, 5 mL of 2.5% TBAF/DMSO solvent and 5 mL of 5.5% TBAF/DMSO solvent were mixed in a 50 mL conical tube to obtain 10 mL of mixed TBAF/DMSO solvent. MCC powder corresponding to half of the total cellulose concentration of 2.5% and CNC powder corresponding to the other half were weighed and added simultaneously. Immediately after powder addition, the mixture was sufficiently vortexed to ensure uniform dispersion of the MCC and CNCs and facilitate their interaction with TBAF/DMSO. The other MCC/CNC solutions were prepared in the same manner. The TBAF/DMSO solvent volume, nominal TBAF concentration, MCC concentration, and CNC concentration for each MCC:CNC compositional ratio are summarized in Table 1.

The prepared MCC/CNC solutions were used immediately for time-dependent viscosity measurement or regenerated MCC/CNC hydrogel fabrication. To fabricate the regenerated MCC/CNC hydrogels, the cellulose solutions with different MCC:CNC compositional ratios were cast into molds and maintained at room temperature overnight to allow the formation of self-supporting gels. Gel formation was identified by the loss of flowability upon inversion. Subsequently, the formed gels were directly immersed in distilled water for cellulose regeneration and solvent exchange. During this process, the distilled water was replaced repeatedly until no visible hazy appearance associated with DMSO–water exchange was observed. The disappearance of the visible hazy appearance was utilized as a practical indication of solvent exchange. Residual TBAF and DMSO were not independently quantified in this study; however, a similar water-exchange procedure used in our previous study was shown to effectively remove TBAF/DMSO, as verified by elemental analysis and combustion ion chromatography [19]. The regenerated MCC/CNC hydrogels were stored in a hydrated state before characterization. For FT-IR spectroscopy, XRD, TGA/DTG, and SEM analyses, the hydrogel samples were prepared through freeze-drying.

### 4.3. Characterization of the MCC/CNC Solutions and Regenerated Hydrogels

The viscosity of the cellulose solutions was measured using a digital rotational viscometer (DV2T, AMETEK Brookfield, Middleboro, MA, USA) equipped with an SC4-34 spindle at a shear rate of 1 s^−1^. Each solution was prepared in a 50 mL conical tube using 10 mL of DMSO, and the entire prepared solution was transferred to the measurement chamber. Measurements were performed at ambient temperature, with viscosity values recorded at 5 s intervals. Each condition was measured in triplicate (*n* = 3). The measurements were terminated when the instrument displayed the over-range indication (EEEE). For the individual 2.5% MCC and CNC solutions, the viscosity was measured as a function of TBAF concentration to identify the TBAF concentration that produced the maximum viscosity for each cellulose source. Within each cellulose-source series, viscosity values obtained at a common reference elapsed time were used to compare the different TBAF concentrations. Because the MCC and CNC series exhibited markedly different rates of viscosity increase, the reference elapsed time was selected separately for each series to identify the TBAF concentration associated with the maximum viscosity. For the mixed MCC/CNC formulations, the time-dependent viscosity profiles were recorded at 5 s intervals immediately after preparation until the measurement range of the viscometer was exceeded.

The mechanical properties of the regenerated MCC/CNC hydrogels were evaluated in terms of gel strength and cutting strength using a texture analyzer (Compac-100II, Sun Scientific Co., Ltd., Tokyo, Japan). Gel strength was measured using a flat-ended plunger adapter (No. 14, Ø 50 mm). Cylindrical hydrogel specimens were prepared with a height of 17 mm and a diameter of 25 mm. The test was performed at a crosshead speed of 60 mm/min with a maximum load capacity of 19.6 N. The force–distance curves were recorded during compression, and the gel strength was calculated as the area under the force–distance curve. The results are expressed in N·mm. Cutting strength was measured using a knife-edge adapter (No. 10). Hydrogel samples were prepared as rectangular specimens with a thickness of 0.2 cm, a length of 5.0 cm, and a width of 2.0 cm. The test was conducted at a crosshead speed of 50 mm/min. The force–distance curves were recorded during cutting, and the cutting strength (in N·mm) was calculated as the area under the force–distance curve. For each formulation, the gel strength and cutting strength were measured in triplicate (*n* = 3). The water content of the regenerated MCC/CNC hydrogels was determined gravimetrically. The weight of each hydrated hydrogel (W) was measured, after which the sample was freeze-dried and reweighed to obtain the dry weight (D). The water content was calculated as [(W − D)/W] × 100. The measurements were performed in triplicate (*n* = 3).

The optical properties of the regenerated MCC/CNC hydrogels were evaluated using a UV–Vis spectrophotometer (UV-1280, Shimadzu Corp., Kyoto, Japan). The UV–Vis transmittance spectra were recorded over the wavelength range of 200–800 nm. Additionally, the hydrated hydrogel samples were placed on a printed background and photographed to compare their macroscopic transparency.

The photophysical relaxation behavior of the regenerated MCC/CNC hydrogels was evaluated via TRPL analysis. TRPL measurements were performed using an FLS1000 spectrometer (Edinburgh Instruments Ltd., Livingston, UK) equipped with a time-correlated single-photon counting (TCSPC) module. A 375 nm picosecond pulsed diode laser was used as the excitation source. Emission decay curves were collected at the maximum emission wavelength of each MCC/CNC hydrogel sample. The resulting TRPL decay profiles were fitted using a multi-exponential decay model to calculate the fast lifetime component (τ_1_), amplitude-weighted average lifetime (τ_amp), and fractional contribution of the fast decay component (B_1_). The TRPL parameters were obtained from a single measurement for each formulation (*n* = 1) and were interpreted descriptively.

The chemical structure of the regenerated MCC/CNC hydrogels was analyzed using FT-IR spectroscopy. The FT-IR spectra were acquired using a Frontier FT-IR spectrophotometer (PerkinElmer Inc., Waltham, MA, USA) equipped with an ATR accessory over the range of 400–4000 cm^−1^ at a resolution of 4 cm^−1^ across 64 scans.

The XRD patterns shown in Figure S1 were replotted from the original raw XRD data of the same starting MCC and CNC materials previously reported in Refs. [19] and [3], respectively. The original data were acquired in separate measurement sessions using the same SmartLab X-ray diffractometer (Rigaku Corp., Akishima, Japan) under the same measurement conditions. The scans were collected at 2°/min over the 2θ range of 10–40°. The regenerated MCC/CNC hydrogels were analyzed using an Empyrean diffractometer (PANalytical BV, Almelo, The Netherlands) with Cu Kα radiation (λ = 1.5418 Å) over the same 2θ range of 10–40° at a scan rate of 2°/min. The crystallinity index (CrI, %) of the starting MCC and CNC materials was calculated as CrI = [(Icr − Iam)/Icr] × 100, where Icr is the maximum intensity of the main crystalline diffraction peak near 2θ = 22.6° and Iam is the minimum intensity in the amorphous region near 2θ = 18–19°, as previously described [9]. Since different diffractometers were used for the starting materials and regenerated hydrogels, the two datasets were compared qualitatively rather than through a direct quantitative comparison of crystallinity indices.

The thermal degradation behavior of the regenerated MCC/CNC hydrogels was analyzed using TGA and DTG. TGA was performed using a Q500 thermogravimetric analyzer (TA Instruments, New Castle, DE, USA) under a nitrogen atmosphere. Samples were heated from 30 to 700 °C at a heating rate of 10 °C/min, and the nitrogen flow rate was set to 100 mL/min.

The morphology of the freeze-dried regenerated MCC/CNC hydrogels was examined using a field-emission scanning electron microscope (SU8600, Hitachi High-Tech Corp., Tokyo, Japan). The samples were sputter-coated with a thin layer of platinum prior to observation. Surface morphologies were observed at an accelerating voltage of 5 kV and a magnification of 5000×, whereas cross-sectional morphologies were observed at 5 kV and a magnification of 1000×.

### 4.4. Statistical Analysis

Quantitative data obtained from replicate measurements are presented as mean ± standard deviation. Statistical analysis was performed using the Statistical Package for the Social Sciences (SPSS) software version 21.0 (IBM Corp., Armonk, NY, USA). Differences among samples were analyzed via one-way analysis of variance. Duncan’s multiple range test was used when the homogeneity of variance assumption was satisfied, whereas the Games–Howell test was applied when this assumption was not satisfied. Differences were considered statistically significant at *p* < 0.05.

## Figures and Tables

**Figure 1:**
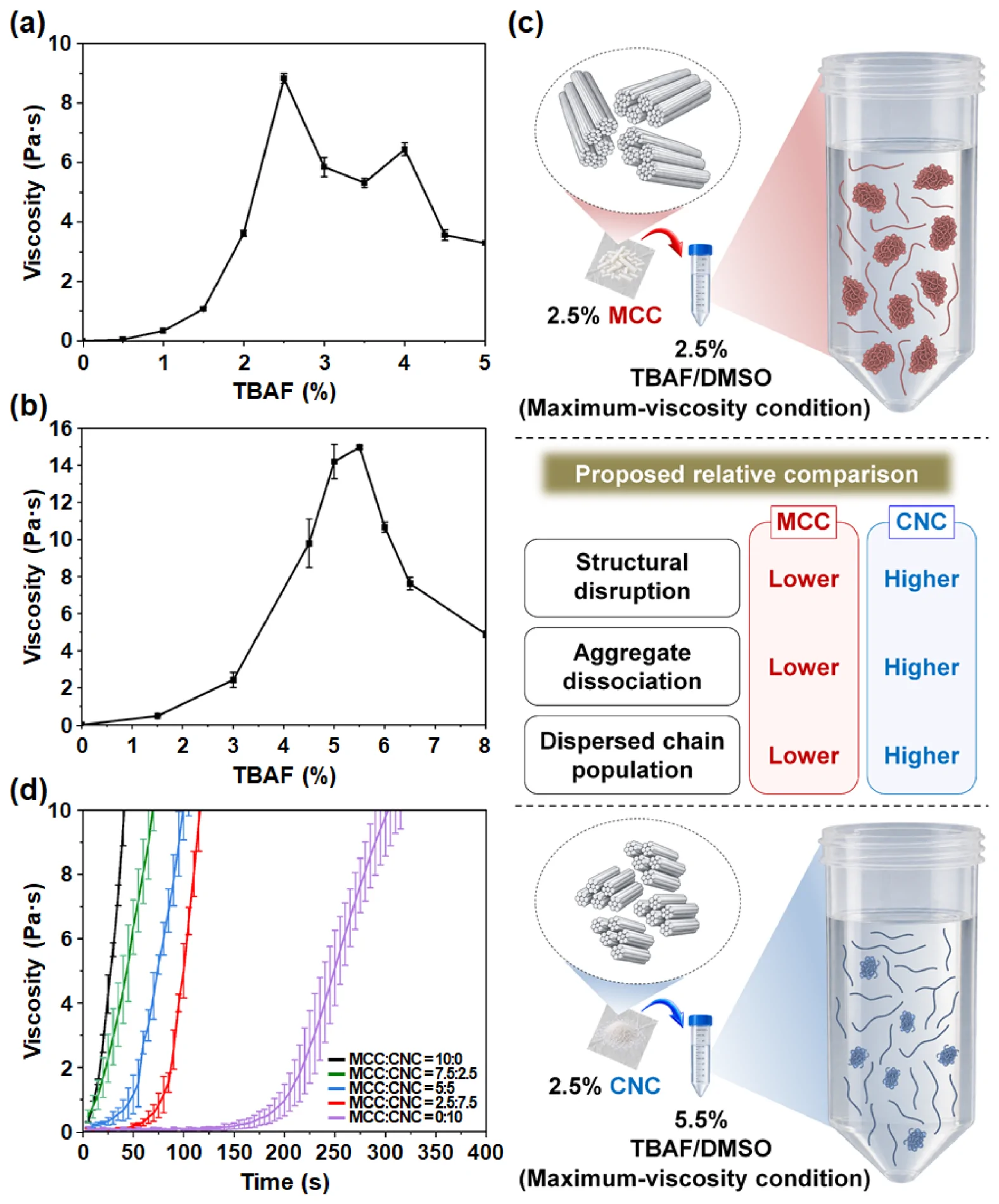
Viscosity behavior of MCC, CNCs, and MCC/CNC solutions in TBAF/DMSO. (**a**) Viscosity of 2.5% MCC solutions prepared in TBAF/DMSO with different TBAF concentrations. (**b**) Viscosity of 2.5% CNC solutions prepared in TBAF/DMSO with different TBAF concentrations. (**c**) Proposed schematic comparison of the relative differences in structural disruption, aggregate dissociation, and dispersed chain population in the MCC and CNC solutions under their respective maximum-viscosity TBAF concentrations (2.5% for MCC and 5.5% for CNCs). (**d**) Time-dependent viscosity changes of MCC/CNC solutions prepared in TBAF/DMSO at different MCC:CNC ratios. The viscosity measurements in (**a**,**b**,**d**) were acquired at a shear rate of 1 s^−1^, and the values are presented as mean ± standard deviation.

**Figure 2:**
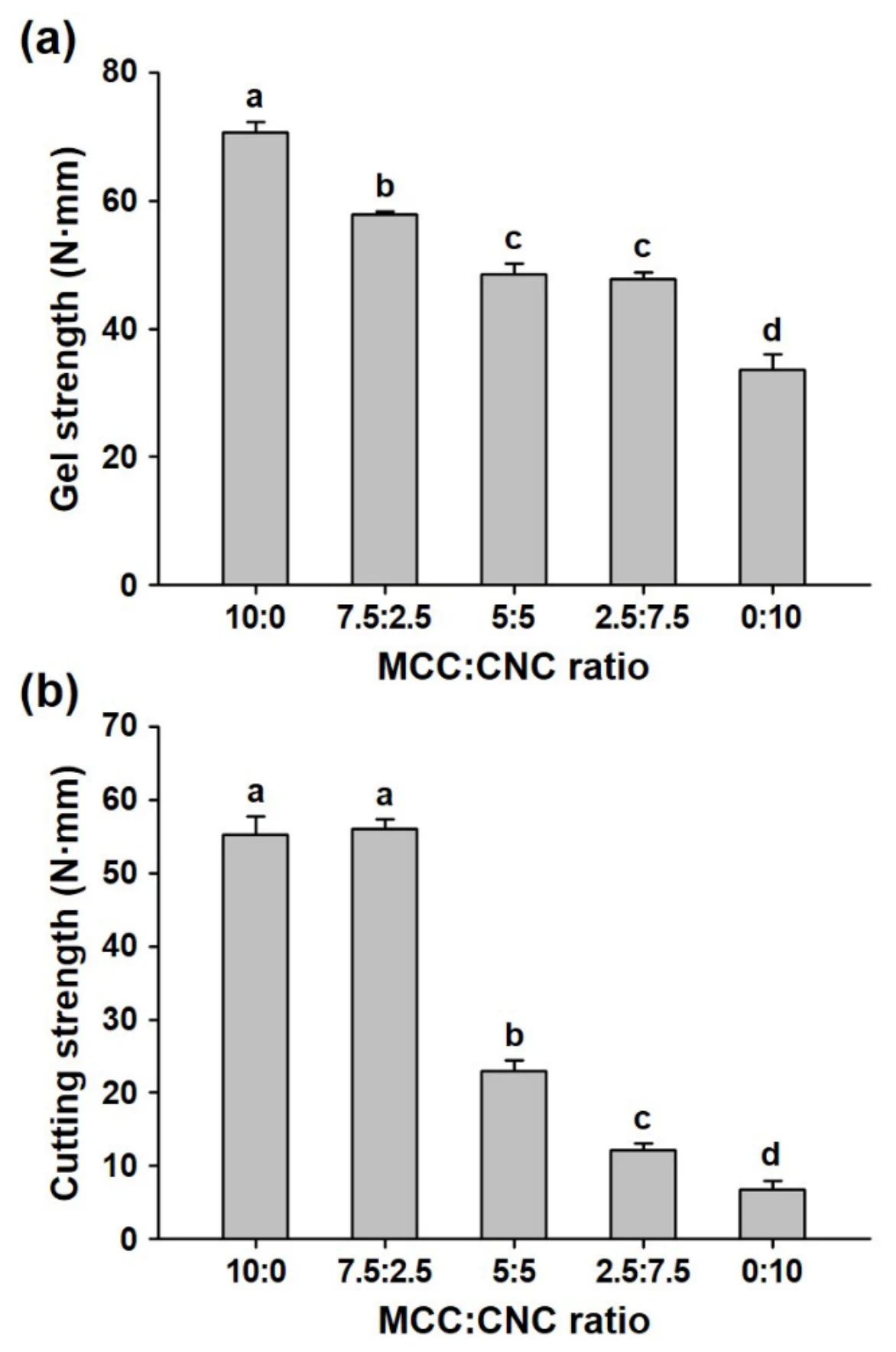
Mechanical properties of the regenerated MCC/CNC hydrogels. (**a**) Gel strength and (**b**) cutting strength of the regenerated MCC/CNC hydrogels with different MCC:CNC ratios. The different letters indicate significant differences among samples (*p* < 0.05).

**Figure 3:**
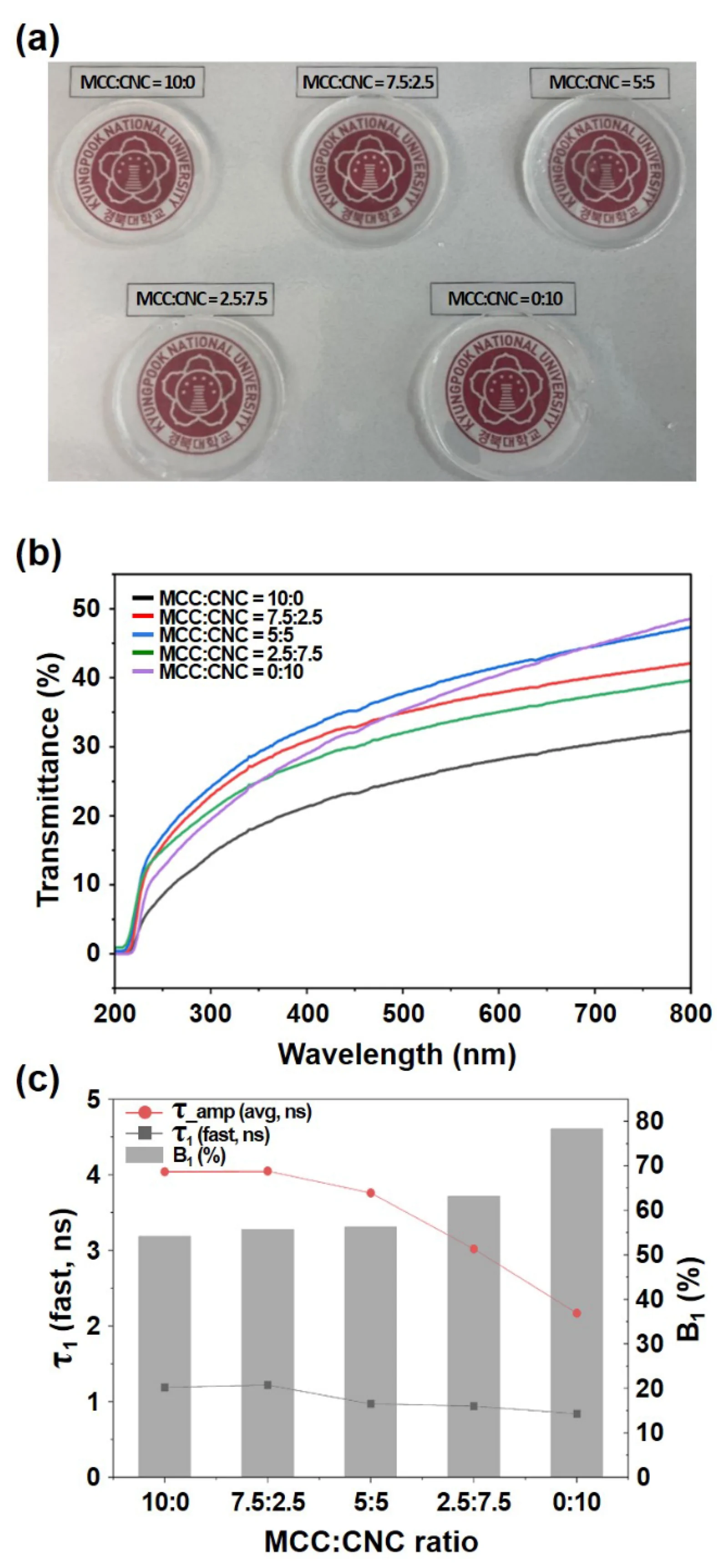
Optical and photophysical properties of the regenerated MCC/CNC hydrogels. (**a**) Photographs of the regenerated MCC/CNC hydrogels placed on a printed background, (**b**) UV–Vis transmittance spectra, and (**c**) TRPL parameters of the regenerated MCC/CNC hydrogels with different MCC:CNC ratios. τ_1_, τ_amp, and B_1_ represent the fast lifetime component, amplitude-weighted average lifetime, and fractional contribution of the fast decay component, respectively.

**Figure 4:**
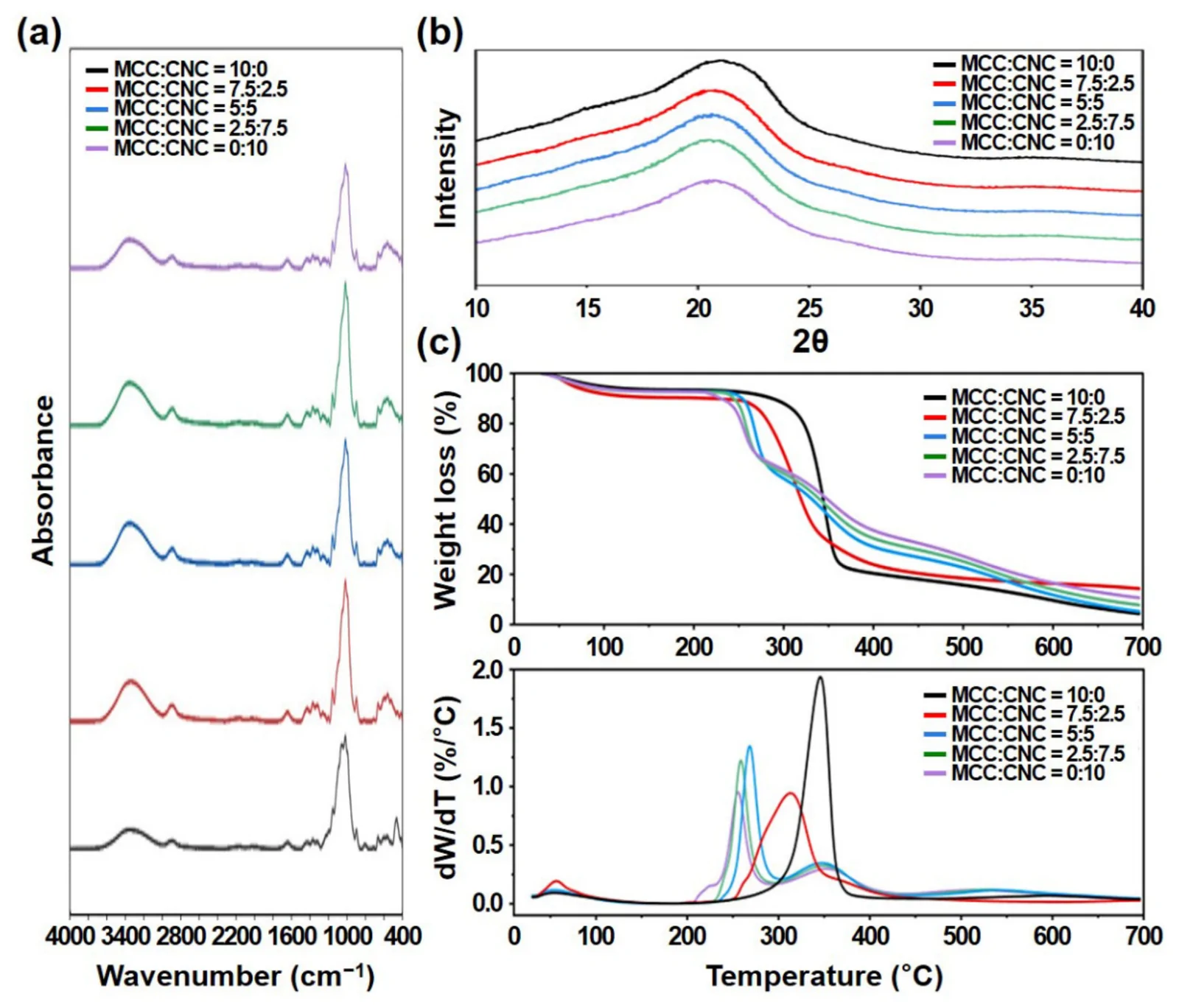
Chemical, structural, and thermal characterization of the regenerated MCC/CNC hydrogels. (**a**) FT-IR spectra, (**b**) XRD patterns, and (**c**) TGA curves and DTG profiles of the regenerated MCC/CNC hydrogels with different MCC:CNC ratios.

**Figure 5:**
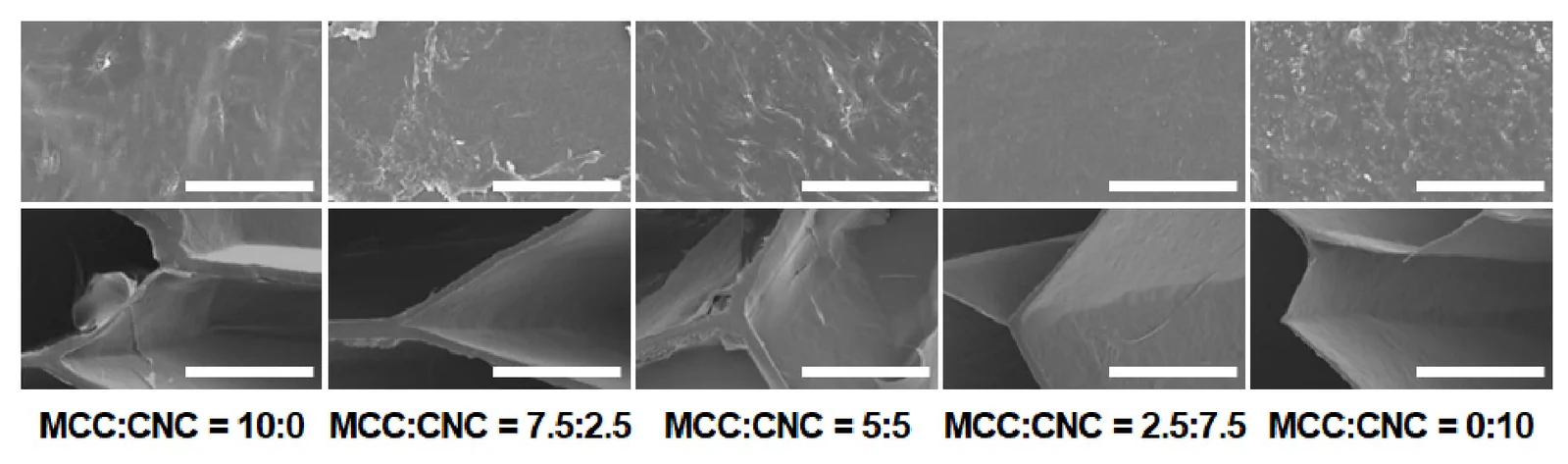
SEM images of freeze-dried regenerated MCC/CNC hydrogels at different MCC:CNC ratios. Top row: surface morphologies at 5000× magnification (scale bars = 10 μm). Bottom row: cross-sectional morphologies at 1000× magnification (scale bars = 50 μm).

**Table 1:** Formulation matrix of the MCC/CNC solutions prepared in TBAF/DMSO for regenerated hydrogel fabrication.

Parameter	MCC:CNC Ratio				
	10:0	7.5:2.5	5:5	2.5:7.5	0:10
2.5% TBAF/DMSO volume (mL)	10.0	7.5	5.0	2.5	0.0
5.5% TBAF/DMSO volume (mL)	0.0	2.5	5.0	7.5	10.0
Nominal TBAF concentration (%)	2.50	3.25	4.00	4.75	5.50
MCC concentration (%)	2.500	1.875	1.250	0.625	0.000
CNC concentration (%)	0.000	0.625	1.250	1.875	2.500
Total cellulose concentration (%)	2.5	2.5	2.5	2.5	2.5

## Data Availability

The original contributions presented in this study are included in the article. Further inquiries can be directed to the corresponding author.

## Supplementary Materials

The following supporting information can be downloaded at: https://www.mdpi.com/article/10.3390/gels12100878/s1, Figure S1: XRD patterns of the starting MCC and CNC materials used in this study. The MCC and CNC patterns were replotted from the original raw XRD data previously reported in Refs. [19] and [3], respectively. The crystallinity indices calculated from the respective patterns were 73% for MCC and 70% for CNC.; Table S1: Water content of regenerated MCC/CNC hydrogels with different compositional ratios.

## Abbreviations

The following abbreviations are used in this manuscript:

MCC	Microcrystalline cellulose
CNCs	Cellulose nanocrystals
TBAF	Tetrabutylammonium fluoride
DMSO	Dimethyl sulfoxide
DP	Degree of polymerization
DTG	Derivative thermogravimetry
FT-IR	Fourier-transform infrared
TGA	Thermogravimetric analysis
TRPL	Time-resolved photoluminescence
UV–Vis	Ultraviolet–visible
XRD	X-ray diffraction
