# Supplementary material for: Composition-Dependent Viscosity and Properties of MCC/CNC Regenerated Cellulose Hydrogels Prepared Using TBAF/DMSO
Source: Gels. 2026 Sep 28;12(10):878. doi: 10.3390/gels12100878 (PMC13703987; doi:10.3390/gels12100878)
Supplement: Supplementary file 1 [file gels-12-00878-s001.zip › gels-4539009-supplementary.pdf]

Composition-Dependent Viscosity and Properties of MCC/CNC Regenerated Cellulose Hydrogels Prepared Using TBAF/DMSO

Na Hyeon Kim[1], Maria Jose Silva Pincay[1], Yeongjun Kim[1], Hyosub Kim[1], Eunok Jang[1], Dong Hyun Kim[1], Sae-Byuk Lee[1], Mi-Kyung Park[1], Seockmo Ku[2], Eunsook Lee[3], and Deokyeong Choe [1,*]

[1]School of Food Science and Biotechnology, Kyungpook National University, Daegu 41566, Republic of Korea

[2]Department of Food Science and Technology, Texas A&M University, College Station, TX 77843, USA

[3]Department of Pharmaceutical Science, Florida A&M University, Tallahassee, FL 32307, USA

***Address correspondence to:**

Deokyeong Choe, Ph.D., E-mail: cd02da@knu.ac.kr

Supplementary data

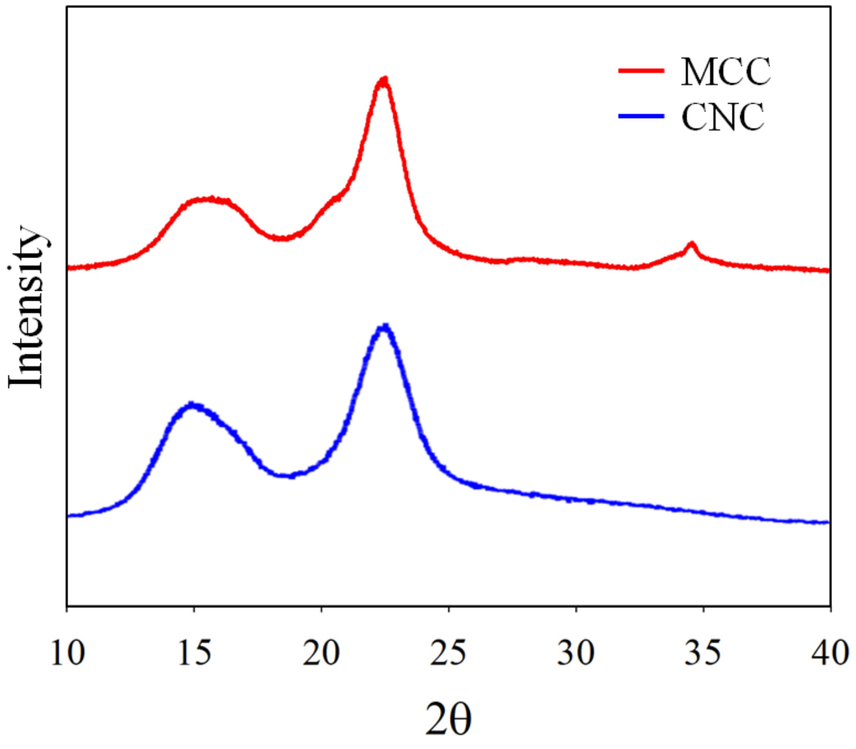

Figure S1. XRD patterns of the starting MCC and CNC materials used in this study. The MCC and CNC patterns were replotted from the original raw XRD data previously reported in Refs. [19] and [3], respectively. The crystallinity indices calculated from the respective patterns were 73% for MCC and 70% for CNC.

Table S1. Water content of regenerated MCC/CNC hydrogels with different compositional ratios

Samples	Water content (%)
MCC:CNC = 10:0	95.22 ± 0.05^{e}
MCC:CNC = 7.5:2.5	96.18 ± 0.05^{d}
MCC:CNC = 5:5	96.77 ± 0.21^{c}
MCC:CNC = 2.5:7.5	97.42 ± 0.20^{b}
MCC:CNC = 0:10	97.97 ± 0.19^{a}

Values are presented as mean ± standard deviation (n = 3). Different superscript letters indicate significant differences among samples ($p < 0.05$).
